# Research progress of Bub3 gene in malignant tumors

**DOI:** 10.1002/cbin.11740

**Published:** 2022-02-24

**Authors:** Chenyang Wang, Dating Cheng, Chenglong Pan, Chunyan Wang, Zhi Nie

**Affiliations:** ^1^ Department of Pathology First Affiliated Hospital of Kunming Medical University Kunming Yunnan China

**Keywords:** Bub3, malignant tumors, spindle assembly checkpoint gene

## Abstract

The spindle assembly checkpoint (SAC) is a highly conserved monitoring system that ensures a fidelity of chromosome segregation during mitosis. Bub3, a mitotic Checkpoint Protein, is a member of the Bub protein family, and an important factor in the SAC. Abnormal expression of Bub3 results in mitotic defects, defective spindle gate function, chromosomal instability and the development of aneuploidy cells. Aneuploidy is a state of abnormal karyotype that has long been considered as a marker of tumorigenesis. Karyotypic heterogeneity in tumor cells, known as “chromosomal instability” (CIN), can be used to distinguish cancerous cells from their normal tissue counterpart. In this review, we summarize the expression and clinical significance of Bub3 in a variety of tumors and suggest that it has potential in the treatment of cancer.

## HIGHLIGHT

The spindle assembly checkpoint (SAC) is an evolutionarily highly conserved mitotic monitoring system that ensures a high fidelity of chromosomes during mitosis. Bub3 Mitotic Checkpoint Protein is a member of the Bub family and is an important component of the SAC. Abnormal expression of Bub3 results in defective spindle gate function, chromosomal instability and the development of aneuploidy cells. Aneuploidy, which represents a “state” of abnormal karyotype, has long been recognized as a hallmark of tumorigenesis (Bakhoum et al., 2017). Karyotypic heterogeneity in tumor cells draws its origin from “chromosomal instability” (otherwise known as CIN), a hallmark that distinguishes most cancers from its normal tissue counterpart (Lengauer et al., 1998; Bakhoum et al., 2017). In the process of cell mitosis, it is inevitable to be damaged by various physical, chemical, biological and other external factors. Various kinds of damage detection and repair gene regulation are critical to avoid abnormal cell division (Chao W. C. et al., 2012). Bub3 protein is an important component of the SAC during cell mitosis, which can maintain accurate chromosomal segregation and prevent the formation of aneuploidy during mitosis. Abnormal expression of Bub3 in a variety of malignancies suggests that Bub3 plays an important role in the genesis, progression, and metastasis of malignancies. This article will discuss the research progress of Bub3 in malignant tumors.

**Abbreviations**
CIN: chromosomal instability
SAC: spindle assembly checkpoint
MCC: mitotic checkpoint complex
APC/C: anaphase‐promoting complex/cyclosome
BUB1: BUB1 mitotic checkpoint serine/threonine kinase
BUB3: BUB3, mitotic checkpoint protein
BUBR1/BUB1B: BUB1 mitotic checkpoint serine/threonine kinase B
K‐MT: Kinetochore‐microtubule
OSCC: oral squamous cell carcinoma
OLP: oral lichen planus
ISOC: in situ oral carcinoma
CNS: entral nervous system
CRC: colorectal cancer
HCC: hepatocellular carcinoma
ACC: adrenocortical carcinoma
NSCLC: nonsmall cell lung cancer
PDAC: pancreatic ductal adenocarcinoma
IHC: immunohistochemistry
TNBC: triple negative breast cancer
MVA: mosaic variegated aneuploidy
ER: estrogen receptor
PR: progesterone receptor
HER2: human epidermal growth factor

## 1 INTRODUCTION

Bub3 is an important component of the spindle assembly checkpoint (SAC), which is required to ensure that chromosomal segregation occurs accurately during mitosis. Abnormal expression of Bub3 in a variety of malignancies suggests that it plays an important role in the genesis, progression, and metastasis of malignancies. This article discusses recent research progress into Bub3 in malignant tumors.

## 2 BUB3 OVERVIEW

### 2.1 The SAC and mitotic checkpoint complex (MCC)

The cell cycle is the process by which a cell divides. After chromosome replication, microtubules connect sister chromatids and direct them to the middle of the cell (Primorac et al., 2013). Accurate chromosome segregation relies on bioriented, amphitelic attachments of chromosomes to microtubules of the mitotic spindle, in which the sister chromatids must be attached to opposite spindle poles (Edwards et al., 2018; Primorac et al., 2013). The SAC is activated when improper attachment occurs. A highly complex network of proteins, the SAC ensures the sister chromatids are lined up correctly before the initiation of anaphase (Chao W. C. et al., 2012; Primorac et al., 2013). If the SAC is deficient or incorrect, aneuploidy can occur and cancer can develop. The main components of SAC are the BUB and MAD family members. Among them, Mad2, BubR1, Cdc20 and Bub3 form the soluble MCC which restrains anaphase‐promoting complex/cyclosome (APC/C) activation by targeting Cdc20 (an APC/C co‐activator), resulting in a delay of the metaphase‐anaphase transition until proper kinetochore‐microtubule (K‐MT) attachment are established (Cheeseman & Desai, 2008; Dai et al., 2016; Musacchio & Salmon, 2007; Wei et al., 2010). Upon activation of the SAC by improperly attached chromosomes, Mad1 and Bub1 are recruited to the kinetochores, which causes Mad2 in an open state (O‐Mad2), to convert to an closed state (C‐Mad2). At the same time, Bub1 recruits and stabilizes BubR1 (the complex formed with Bub3) to unattached kinetochores, where BubR1 (the complex formed with Bub3) binds Cdc20 (the complex formed with C‐Mad2) forming the MCC (Breit et al., 2015; hung & Chen, 2002; Nezi et al., 2006; Rashid et al., 2018). Next, the MCC binds the APC in association with its co‐activator, Cdc20. When this happens the APC/C‐bound Cdc20 prevents APC‐substrate recognition, thereby inhibiting the APC/C activity, and preventing anaphase onset (Rashid et al., 2018; Zhou et al., 2016). Once all the chromosomes are correctly aligned at the metaphase plate and are under tension, the SAC is switched off and cdc20 is released, activating APC/C, which then degrades securin and cyclin B, promoting the activation of separase which takes the cohesin complex away from the chromosome and the onset of anaphase (Dai et al., 2016; Morrow et al., 2005; Musacchio & Salmon, 2007; Toledo et al., 2014). This article provides a brief overview of the involvement of Bub3 in relation to the SAC and cancer.

### 2.2 Bub3 overview and related mechanisms in malignancy

The chromosome location of the human Bub3 gene is 10q26 (Kwon TK et al., 2000). Bub3 plays a role both in SAC signaling and in facilitating the establishment of proper K‐MT attachment. Bub3 can promote the formation of stable end‐on bipolar attachments and respectively form complexes with Bub1 and BubR1/Mad3 assisting in the accurate localization of Bub1 and BubR1/Mad3 on the kinetochore. According to studies, kinetochore localization of Bub1 and BubR1 requires phosphorylation by the MELT motifs in the Knl1 (Overlack et al., 2017). Knl1 is part of the KMN network, a very large protein complex present at the kinetochores. When the SAC is activated, Bub3 first binds directly to Knl1 through a region that contains multiple MELT motifs. Once bound to the Knl1, Bub3 recruits Bub1 to the kinetochore and forms a complex with Bub1. BubR1/Bub3 are then recruited to the kinetochore through direct interaction with Bub1 (Primorac et al., 2013). Thus, the kinetochore location of BubR1 depends on Bub3‐dependent Bub1 recruitment. After that, BubR1/Bub3 binds with the MCC inhibiting the ubiquitin ligase activity of the APC/C, thus preventing the destruction of securin and cyclin B thereby inhibiting the cell transition from metaphase to anaphase (Musacchio & Salmon, 2007; Yang & Lacefield, 2016). The MCC inhibits APC/C ubiquitin ligase activity by phosphorylating Cdc20 (Chung E et al., 2003). Studies have shown that APC/Cdc20 binds BubR1 but not Mad2 (Han et al., 2013). Han et al. (2014) found that Bub3 promotes BubR1‐Cdc20 interaction and mediates BubR1‐dependent kinetochore recruitment of Cdc20. The Bub3/Bub1 complex can also bind to telomeres during S phase and promote telomere DNA replication (Li F et al., 2018). Bugz has been shown to interact with Bub3 to stimulate mitotic function by promoting its stability and kinetochore load (Han JS et al., 2014; Shirnekhi et al., 2020). However, how Bub3 locates to the kinetochore, how Bub3 binds to BubR1, and what role it plays in mitosis is still unknown.

In an oral squamous cell carcinoma (OSCC) study, Zheng et al. (2020) mentioned that Spindly and Bub3 may play a synergistic role in accelerating the OSCC process and increasing mortality. Spindly is involved in chromosome alignment and SAC signal transduction, as well as in the regulation of dynein at kinetochores (Chan et al., 2009; Griffis et al., 2007; Silva et al., 2019). However, exactly how Spindly and Bub3 interact is not clear.

In an non‐small cell lung cancer (NSCLC) study, Guo et al. (2021) found that the overexpression of lnc CRYBG3 RNA inhibited the interaction between Cdc20 and Bub3, which activated the APC/C complex prematurely, causing aneuploidy and accelerated development of NSCLC. Long noncoding RNAs are operationally defined as RNA transcripts of >200 nt length that have limited protein‐coding potential (Pei et al., 2018).

**Figure 1:**
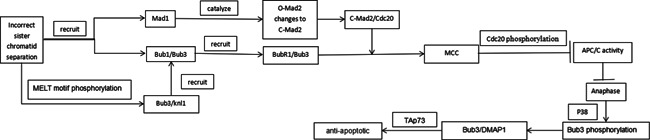
Flow chart of the mechanisms mentioned in the article

The transcription factor p73, a member of the p53 protein family, has TAp73 and ΔNp73 subtypes that regulate cell cycle progression, survival, genomic stability, hypoxia, and angiogenesis (Ozaki & Nakagawara, 2005). The characteristics of the proapoptotic activity of TAP73 are similar to that of the tumour suppressor p53 (Li J et al., 2018). In Vernole et al. (2009) study, they found that Bub3 binds to the TAP73α subtype in tumor cells, promoting aneuploidy formation, increasing faulty cell division and genomic instability, and influencing tumor cell apoptosis, which plays an important role in cancer progression. Interestingly, in pancreatic cancer cells, when mitosis is stopped, p38‐dependent phosphorylation promotes Bub3 binding to DMAP1 (Li J et al., 2018). Their study showed that the phosphorylation of Bub3 S211 mediated by p38 activation was restricted on the presence of mitotic cessation and was not stimulated by transforming growth factor beta during interphase, consistent with the result that p38/Bub3 interaction can be detected only during mitosis. DMAP1 is highly phosphorylated by c‐Src in pancreatic cancer cells. When mitosis arrests, this phosphorylation inhibits the formation of the DMAP1/Bub3 complex, which finally maintains cell survival. By interacting with TAp73, Bub3/DMAP1 enhanced DNMT1‐mediated DNA methylation at the promoter region of antiapoptotic genes, thereby blocking postmitotic gene transcription and leading to increased cellular apoptosis. However, the mechanism by which Bub3 mediates tumorigenesis and progression in tumors remains unclear (Figure 1).

CIN (chromosomal instability) is a common feature of many malignant cells (Breit et al., 2015; Zhang et al., 2018) and both increased and decreased SAC gene expression (Mad2, BUB1) induces aneuploidy and favors tumor development (Simonetti et al., 2019). The loss of Bub3 function results in chromosomal dysregulation and aneuploidy, contributing to cancer evolution (Li et al., 2009; Sansregret & Swanton, 2017). Bub3 has been found to be abnormally expressed in many human cancers, including oral cancer, Glioma tumors, colorectal cancer (CRC) and more.

## 3 ORAL CANCER

### 3.1 Oral squamous cell carcinoma

3.1.1

Head and neck cancer is one of the most common cancers (Capparuccia & Tamagnone, 2009). The number of OSCC cases accounts for more than 90% of head and neck tumors (Tandon et al., 2017). OSCC occurs in the oral cavity and oropharynx and can be caused by a variety of etiological factors. Smoking and alcohol are still the most common risk factors (Graham et al., 1977). Gene mutations may also contribute to the development of cancers in the pharynx and oral cavity (Krishna et al., 2015). In many previous cancer reports, the expression of many SAC components has been altered, and some of these components have been known as suitable targets for cancer therapy (Silva et al., 2011; Barbosa et al., 2011). Silva et al. (2019) used immunohistochemistry to detect Bub3 protein expression in 62 tissue microarrays from OSCC and evaluated the expression of Bub3 in OSCC cell lines and in normal human oral keratinocytes by real‐time PCR. They found that the protein expression level of Bub3 messenger RNA (mRNA) in OSCC was significantly higher than that in adjacent tissues. Moreover, overexpression of Bub3 can serve as an independent prognostic indicator for cancer‐specific survival, and may have an effect on increased cellular proliferation. Zheng et al. (2020) used RT‐PCR to detect the expression of Bub3 mRNA in OSCC and adjacent normal tissues from 65 patients. They also found that the mRNA expression levels of Bub3 in OSCC were significantly higher in adjacent tissues. They then analyzed the collected data and conducted more experiments to find that if the expression of the Bub3 gene was specifically suppressed, the proliferation and migration of cancer cells were reduced. Additionally they reported that the expression of Bub3 mRNA was associated with tumor, node, metastases (TNM) staging, clinical staging and lymph node metastasis. Bub3 mRNA expression was elevated if OSCC tissue was in T3–T4/with lymph node metastasis. The higher the Bub3 mRNA expression level, the lower the 5‐year survival. From these results, it can be indicated that Bub3 can adversely affect prognosis and may play an important role in the development, progression, prognosis and treatment of OSCC and may be involved in the development of OSCC as an oncogenic gene. In addition, If the expression of Bub3 protein is inhibited, it is cytotoxic to OSCC cells and enhanced its chemical sensitivity to cisplatin (Sliva et al., 2019).

### 3.2 Oral lichen planus (OLP) and in situ oral carcinoma (ISOC)

OLP is a relatively common inflammatory mucocutaneous disorder of unknown etiology that mainly occurs in women (Nosratzehi, 2018; Shen et al., 2012). The World Health Organization (WHO) lists OLP as a potential premalignant lesion (van der Meij & van der Waal, 2003). The development of OSCC is one of the most important complications affecting the progression and prognosis of OLP, with a frequency of malignant transformation ranging from 0.4% to 5.3% (Shi et al., 2010). However, the mechanisms of malignant transformation are still unknown. In one OLP case study, 2 months after treatment, histological analysis showed an ISOC. The immunohistochemical results showed strong nuclear Bub3 staining in both OLP and ISOC regions, suggesting that Bub3 may play a similar role in oral cancer and in precursor lesions as OLP (Rosa et al., 2015). However, more scientific research is needed to confirm this hypothesis.

### 3.3 Prostate cancer

Prostate cancer is one of the most common malignancies of male genitourinary system. Ersvær et al. (2020) collected paraffin‐embedded tissue sections from each of the 253 patients treated with radical prostatectomy. Protein levels were determined by immunohistochemistry and they found that cytoplasmic expression of Bub3 protein in 32% of their cases of prostate cancer. In prostate carcinomas, the positive expression of cytoplasmic Bub3 protein was significantly related to recurrence and the mRNA count of BuB3 in the nucleus was weakly related to protein level, neither were related to recurrence. Therefore, the prognostic value of Bub3 protein expression in the cytoplasm in prostate cancer suggests that whether Bub3 protein expression in the cytoplasm has the same or greater significance as other malignancies.

Cadmium is a widely existing pollutant, which has multiple target organs in the human body and a half‐life of 10–30 years in the human body. It is listed as a human Class I carcinogen (Genchi et al., 2020). Several studies have also found cadmium exposure increases the risk of several cancers, including breast cancer, lung cancer, bladder cancer and so on (Satarug et al., 2010). The prostate is a potential carcinogenic organ for cadmium, but its mechanism remains unclear. Guo et al. (2019) downloaded 19 copies of gene chip data from the Gene Expression Omnibus (GEO), including nine prostatic epithelial cell samples exposed to low doses of cadmium and ten cases of normal control. The results of biological function analysis showed that low doses of cadmium can induce genetic changes in normal prostate epithelial cells. One of the differential genes, Bub3, was differentially expressed in prostate epithelial cells treated with low doses of cadmium and was associated with malignant transformation of normal cells. However, further research is needed to confirm this.

### 3.4 Glioma tumors

Glioma tumors are the most common tumor of central nervous system neoplasms, accounting for 81% of malignant brain tumors (Ostrom et al., 2014). The WHO classifies gliomas as Grades I–IV, and their grades correspond to their degrees of malignancy (Wesseling & Capper, 2018). WHO Grade I refers to benign tumors which is assigned to the more circumscribed and have low proliferative potential and occur mainly in childhood and young adults; WHO Grades II–IV tumours have diffuse infiltration and abnormal cell proliferation (diffuse gliomas); Dedifferentiation and mitotic cell activity can also be observed in WHO Grade III tumours; In addition to the characteristics show in the other grades, WHO Grade IV tumours present as pathological proliferation of small vessels and/or necrosis (Alfonso, 2017). Extensive invasion of healthy brain tissue by glioma cells leads to poor prognosis, making it difficult to find curative therapies. Although combined surgery with focal fractionated radiotherapy and the adjuvant temozolomide is a recognized approach for the treatment of glioblastoma, the 5‐year survival of glioblastoma patients is only 5% (Liu et al., 2018). Therefore, further research into the underlying mechanisms of glioma development is needed to identify new diagnostic biomarkers and potential therapeutic targets (Chen et al., 2021). To this end, Bie et al. (2011) conducted quantitative polymerase chain reaction analysis on RNA from 6 normal brain samples and 38 gliomas with survival time data and found that the expression of Bub3 mRNA was associated with tumor grade and prognosis in glioma tumors: with the increase of WHO classification, Bub3 gene expression significantly increased. Bub3 gene was obviously overexpressed in glioblastomas (Grade IV) and its expression increased almost with grade, indicating that Bub3 might serve as a prognostic marker for gliomas. On the basis of this study, Morales et al. (2013) used 8 adult human glioblastoma cell lines and 18 glioblastoma tumor samples for gene expression analysis and found that Bub3 was downregulated in glioblastoma cell lines and tumor samples compared to normal tissues. Bie and Morales's research on Bub3 in GBM has completely opposite results. The reasons for these differences between the two studies remain unknown, and additional but could be due to small sample sizes can be considered for further studies.

### 3.5 Colorectal cancer

The role of Bub3 in common cancer has been the subject of debate over the past few years. de Voer et al. (2013) conducted whole‐exome sequencing on 33 patients with early‐stage CRC and found an additional mutation in Bub3 (patient 1, p.Phe264Leu). The 33 patients include 10 Dutch and 23 Chinese. They then conducted targeted copy number and mutation screening of a replication cohort of 146 patients with familial or early‐onset CRC patients, and 28 unsequenced samples from the initial cohort (*n* = 39) and found 2 missense variants in Bub3 which were not present in 1154 controls and were assumed to be pathogenic. One of these variants (patient 2, p.Lys21Asn) was found to be at the core of the protein, and the second variant (patient 3, p.Arg149Gln) was expected to destabilize the interaction domain of one of the proteins. Patients 1 and 2 showed varying degrees of variegated aneuploidy and structural abnormalities. They believed that these results indicated that mutations or haploinsufficiency of Bub3 lead to mosaic variegated aneuploidy (MVA) and an increased risk of CRC at a young age (Mur et al., 2018). MVA is a rare recessive condition characterized by widespread abnormalities in chromosome number (aneuploidy). Mutations in BubR1, one of the SAC, have also been reported to lead to MVA in families with embryonal rhabdomyosarcoma (Hanks et al., 2004). Broderick et al. (2017) performed an analysis of sequencing data on 863 cases of familial CRC and 1604 controls. All cases were diagnosed with age ≤55 years and were known to be negative for CRC gene mutations. They found no increase in the frequency of mutations in Bub3 gene in cases compared to controls. Mur et al. (2018) sequenced Bub3 in 456 familial CRC cases and in 88 polyposis cases and found Bub3 c.77C>T (p.T26I) in patient diagnosed with prostate cancer at age 70 and 2 cases of synchronous CRC and 22 cases of adenomatous polyps at age 73. The variant located in the WD40 repeat 1 of a 7‐bladed beta‐propeller fold was expected to be functionally harmful and unstable. In other cancer‐affected family members, the results of co‐segregation did not support a causal role of the variant in cancer aggregation in the family. So, Mur et al. (2018) thought that the relatively small number of functionally related mutations identified in familial and/or early onset series does not indicate thatthe Bub3 test should be included in routine genetic diagnostics of familial CRC. To date, 4 CRC families with different novel/rare germline mutations of Bub3 have been identified. However, functional effects of the Bub3 variant have been confirmed, but its causal relationship in the occurrence of CRC, either alone or in combination with other mutations/variants in other genes, remains to be demonstrated (Mur et al., 2018; de Voer et al., 2013; de Voer et al., 2011).

### 3.6 Breast cancer

Breast cancer is one of the most common malignant tumors in women and it can seriously threatens a woman's life and health. Clinically, breast cancer is routinely categorized into four molecular subtypes according to the presence of estrogen receptor (ER), progesterone receptor (PR) and human epidermal growth factor (HER2). Triple negative breast cancer (TNBC) is one of the four types which lacks the expression of ER, PR and HER2 and is characterized by easy metastasis and recurrence, high degree of malignancy and rapid proliferation (Ahmad, 2019; Jhan & Andrechek, 2017). Yuan et al. (2006) quantitatively measured transcripts of the Bub3 gene in the 12 breast cancer cell lines, in “control” breast epithelial cell lines with a fully mitotic spindle injury response (MCF10A and primary mammary epithelial cells), and in high‐grade breast cancers tissue samples. They found that the mRNA and protein levels for Bub3 gene in the genetically unstable mammary cancer cell lines and high‐grade primary breast cancer tissues were significantly higher than that in the stable MCF‐10A and normal breast epithelial cells or in normal breast tissues. So, Bub3 is speculated to be a potential marker for breast cancer. Mukherjee et al. (2015) performed immunohistochemical staining of Bub3 on the nottingham tenovus primary breast cancer series (*n* = 1858) microarrays and found that Bub3 was a key kinase in low‐grade luminal tumours. In low‐grade breast cancers, high protein expression rates of Bub3 was related to longer overall survival, while lower protein expression lead to poorer prognosis. Turner et al. (2010) performed high‐resolution microarray‐based comparative genomic hybridisation on 56 TNBCs, of which 24 were analyzed for genome‐wide gene expression and found that in TNBC, only a few genes including Bub3 located in the repeat region were consistently overexpressed during amplification and could constitute as potential therapeutic targets.

### 3.7 Hepatocellular carcinoma (HCC)

Liver cancer is a malignant tumor, and the degree of malignancy is very high. According to global cancer statistics, about 841,000 new cases of liver cancer patients are diagnosed and 782,000 patients die of the disease annually (Bray et al., 2018). HCC, which accounts for 75%–85% of all liver cancer cases, is the most common histological type of primary liver cancer. Liping et al. (2020) analyzed a large number of HCC data from the GEO and TCGA database and found that Bub3 gene expression level was significantly related to mortality, and patients with a higher expression level of Bub3 gene were expected to have a poorer prognostic outcome. It is speculated that Bub3 is a negative prognostic biomarker for HCC patients.

### 3.8 Adrenocortical carcinoma (ACC)

ACC is a rare malignant tumor with high degree of malignancy and low survival rate, but it is the most common primary cancer in the adrenal gland (Chandrasekar et al., 2019). Despite the recent discovery of several important molecular pathways related to its aggressiveness and biology, the prognosis of advanced ACC is still poor and novel treatments are needed (Lam, 2021; Subramanian & Cohen, 2019). Subramanian and Cohen (2019) analyzed TCGG and other databases and found that Bub3 gene was significantly upregulated in Stage IV of ACC and was correlated with poorer survival. More studies are needed to confirm this conclusion.

### 3.9 Lung tumor

Lung cancer is a malignant tumor with high mortality. NSCLC is the most common type of lung cancers in human and has one of the highest mortality and morbidity rates in China (Bray et al., 2018) and the rest of the world (Guo et al., 2021; Siegel et al., 2013). In lung cancer with chromosomal instability, mitotic checkpoint dysfunction is often found. Kang et al. (2017) evaluated the impact of 73 SNPs in Bub3 gene on the survival outcomes in patients with surgically resected NSCLC and discovered that the expression of Bub3 mRNA in lung tumor tissues was higher than that in nonmalignant lung tissues and patients with the Bub3 rs7897156TT genotype had worse overall survival. In their in vitro luciferase assay, the rs7897156C‐to‐T change increased Bub3 promoter activity and Bub3 mRNA expression of rs7897156CT or TT genotypes was significantly higher than that of rs7897156CC genotype, suggesting that Bub3 rs7897156C>T may be a functional SNP and changes in rs7897156C‐to‐T may lead to overexpression of Bub3 and may effect mitotic checkpoint function, thereby affecting the prognosis of patients with NSCLC. But, more studies are needed to confirm the effect of Bub3 in lung cancer. Guo et al. (2021) found that the expression level of Bub3 gene was highly related to the incidence of NSCLC and positively correlated with TNM staging by using the Cancer Genome Atlas database. The higher the Bub3 gene expression, the lower the survival rate and the higher the metastatic rate. But the exact mechanism is not clear.

### 3.10 Gastric cancer

As one of the most common malignant tumors of the digestive system, gastric cancer has a high mortality rate (Recio‐Boiles & Babiker, 2020). The high mortality rate of gastric cancer, which is mainly due to its advanced‐stage diagnosis, so early detection and treatment are very important (Ma et al., 2021). Therefore, the search for new biomolecules and signaling pathways may provide possible treatment for gastric cancer (Guo et al., 2021). Grabsch et al. (2003) used RT‐PCR to study the expression of the mitotic checkpoint genes BUB1, BUBR1, and Bub3 in 43 gastric cancer tissue samples, and compared them with the expression of non‐neoplastic gastric mucosa in the same patient. They found that the level of Bub3 mRNA was overexpressed in 79% of gastric cancers and the high expression of Bub3 gene was also related to the high expression of Ki67 in GC. In 60.5% of the cases, all three genes were expressed at higher levels in the same tumour tissue than in corresponding normal gastric tissue. In 11.6% of the cases, all three genes were expressed at low levels in the same tumour tissue. They suspected that these results may indicate that a common mechanism may be involved in regulating the expression of the Bub1, Bub1, and Bub3 genes, but the exact mechanism is unknown. Therefore, more studies on the regulation mechanism of Bub3 gene expression in gastric cancer are needed.

### 3.11 Pancreatic cancer

Pancreatic cancer is a kind of tumor with high malignant degree, high morbidity and high mortality. Ductal adenocarcinoma of the pancreas and its subtypes are the most common pancreatic tumors, accounting for 85%–90% of pancreatic tumors. Early metastasis and resistance to anticancer therapy leading to overall poor prognosis are characteristic of pancreatic ductal adenocarcinoma (PDAC). In the metastatic setting, despite improvements in chemotherapy regimens, outcomes remain poor, with a 5‐year survival rate of only 3%. Moreover, no targeted therapy is yet approved for PDAC and immune checkpoint inhibitors are also considered ineffective for PDAC (Kabacaoglu et al., 2018; Piffoux et al., 2021; Waters & Der, 2018). Therefore, we need more and more in‐depth studies to understand the biological mechanism of the occurrence and development of pancreatic cancer and to provide better guidance for its screening and treatment. Shindo et al. (2017) extracted DNA from all samples and sequenced the Bub3 gene. The samples included 854 patients with pancreatic ductal adenocarcinoma and 288 patients with other pancreatic and periampullary neoplasms and 51 patients with non‐neoplastic diseases who underwent pancreatic resection. They found that 33 (3.9%) of the 854 pancreatic ductal adenocarcinoma patients had an identifiable harmful germline mutation, including two patients had a mutation in Bub3 gene without biallelic inactivation. The significance of the Bub3 mutation is not clear and need more research (Table 1).

**Table 1:** Summary of the Bub3 alterations in malignant tumors

Cancer type	Finding	References
OSCC	Bub3 was overexpressed and IHC staining was mainly nuclear in epithelial cells.	Silva et al. (2019); Zheng et al. (2020)
	Specific inhibition of Bub3 gene expression reduced proliferation and migration of cancer cells.	
	The higher the Bub3 mRNA expression level, the lower the 5‐year survival. Higher histological grade and lymph node metastasis resulted in increased Bub3 gene expression.	
	Inhibition of Bub3 protein expression was cytotoxic to OSCC cells and enhanced its chemical sensitivity to cisplatin	Sliva et al. (2019)
OLP and ISOC	Strong nuclear Bub3 staining	Rosa et al. (2015)
Prostate cancer	Cytoplasmic expression of Bub3 was associated with recurrence.	Ersvær et al. (2020)
	The mRNA count of BuB3 in the nucleus was weakly related to protein level, neither were related to recurrence.	
	Bub3 was differentially expressed in prostate epithelial cells treated with low doses of cadmium and was associated with malignant transformation of normal cells.	Guo et al. (2019)
Glioma tumors	The expression of Bub3 mRNA was downregulated and associated with tumor grade and prognosis in glioma tumors.	Bie et al. (2011); Morales et al. (2013)
CRC	Mutations in Bub3: patient 1, p.Phe264Leu; patient 2, p.Lys21Asn; patient 3, p.Arg149Gln	de Voer et al. (2013)
	Bub3 c.77C>T (p.T26I) variant	Broderick et al. (2017)
Breast cancer	The level of Bub3 protein and mRNA were overexpressed in the genetically unstable mammary cancer cell lines and high‐grade primary breast cancer tissues.	Yuan et al. (2006)
	In low‐grade breast cancers, high expression rates of Bub3 protein was related to longer overall survival, while lower expression lead to poorer prognosis.	Mukherjee et al. (2015)
	In TNBC, Bub3 gene located in the repeat region were consistently overexpressed during amplification.	Turner et al., (2010)
HCC	Bub3 gene expression level was significantly related to mortality.	Liping et al. (2020)
	The higher the level of Bub3, the worse the prognosis.	
ACC	Bub3 gene was significantly upregulated in stage IV of ACC and was correlated with poorer survival.	Subramanian and Cohen (2019)
Lung tumor	The level of Bub3 mRNA was overexpressed and patients with the Bub3 rs7897156TT genotype had worse OS.	Kang et al. (2017)
	The expression level of BuB3 gene was positively correlated with TNM stage and metastasis rate, and negatively correlated with survival rate.	Guo et al. (2021)
Gastric cancer	The level of Bub3 mRNA was overexpressed and the high expression was related to the high expression of Ki67.	Grabsch et al. (2003)
	In the same tumor tissue, the expression levels of BUB1, BUB1, and Bub3 gene showed the same trend.	
Pancreatic cancer	Mutation in Bub3 gene without biallelic inactivation (nucleotide change:c.576+1G>A).	Shindo et al. (2017)

Abbreviations: IHC, immunohistochemistry; mRNA, messenger RNA; OSCC, oral squamous cell carcinoma; TNM, tumor, node, metastases.

## 4 CONCLUSION

As a SAC protein, Bub3 restrains instability and aneuploidy, but if mutated problems can arise. Mutations or deletions in Bub3 have been found in CRC, osteosarcoma (Mendoza et al., 2005) and other tumors. The abnormal expression of Bub3 gene in a large number of tumors is closely associated with the regulation of tumor cell proliferation, apoptosis, and the cell cycle, and it also plays an important role in the occurrence, progression, metastasis, and prognosis of tumors. Warren et al. (2002) speculated that compensatory mechanisms caused by functional defects in other mitotic checkpoints may lead to overexpression of mitotic checkpoint genes. In addition, overexpression of mitotic checkpoint genes may lead to defects in mitotic checkpoint function. Currently, there is not much research on how Bub3 promotes tumor development. However, in terms of the expression of Bub3 in multiple malignancies and its effect on malignancies, more in‐depth studies are needed to further explore the role of Bub3 in tumors and its related mechanisms and pathways, which will also help to find new therapeutic targets and improve the prognosis of patients with cancer.

## FUNDING INFORMATION

This study was supported in part by grants from Department of Science and Technology of Yunnan Province‐Kunming Medical University (2019FE001(‐063) to Wang, C), Yunnan Health Training Project of High Level Talents (D2018058 to Wang, C).

## CONFLICT OF INTERESTS

The authors declare that there are no conflict of interests.

## Data Availability

Data is available.
